# Genetic Diversity of Koala Retroviral Envelopes

**DOI:** 10.3390/v7031258

**Published:** 2015-03-17

**Authors:** Wenqin Xu, Kristen Gorman, Jan Clement Santiago, Kristen Kluska, Maribeth V. Eiden

**Affiliations:** Section on Directed Gene Transfer, Laboratory of Cellular and Molecular Regulation, National Institute of Mental Health, National Institutes of Health, Bethesda, MD 20892, USA; E-Mails: xuwenqin@mail.nih.gov (W.X.); klgorman11@yahoo.com (K.G.); janclement.santiago@nih.gov (J.C.S.); kristen.kluska@nih.gov (K.K.)

**Keywords:** koala retrovirus (KoRV), endogenous retrovirus (ERV), exogenous retrovirus, recombination

## Abstract

Genetic diversity, attributable to the low fidelity of reverse transcription, recombination and mutation, is an important feature of infectious retroviruses. Under selective pressure, such as that imposed by superinfection interference, gammaretroviruses commonly adapt their envelope proteins to use alternative receptors to overcome this entry block. The first characterized koala retroviruses KoRV subgroup A (KoRV-A) were remarkable in their absence of envelope genetic variability. Once it was determined that KoRV-A was present in all koalas in US zoos, regardless of their disease status, we sought to isolate a KoRV variant whose presence correlated with neoplastic malignancies. More than a decade after the identification of KoRV-A, we isolated a second subgroup of KoRV, KoRV-B from koalas with lymphomas. The envelope proteins of KoRV-A and KoRV-B are sufficiently divergent to confer the ability to bind and employ distinct receptors for infection. We have now obtained a number of additional KoRV envelope variants. In the present studies we report these variants, and show that they differ from KoRV-A and KoRV-B envelopes in their host range and superinfection interference properties. Thus, there appears to be considerable variation among KoRVs envelope genes suggesting genetic diversity is a factor following the KoRV-A infection process.

## 1. Introduction

A retrovirus has been implicated in the lymphoid neoplasias that have been plaguing koalas for the last several decades [1,2]. In 2000, a group of Australian scientists isolated, characterized, and sequenced a gammaretroviral provirus present in feral and captive koalas [3]. This retrovirus, now designated KoRV-A has several distinctive features, one being that it is an endogenous retrovirus [4]. However, unlike most endogenous retroviruses that entered the germ line millions of years ago and, over time, were rendered defective and non-pathogenic, KoRV-A is infectious [3,5]. Furthermore, although KoRV-A is an endogenous retrovirus, it is not present in all koala genomes. It appears to be spreading through the koala population from koalas in the north to those living in the south [6,7].

As KoRV-A is transitioning from an exogenous to an endogenous form, it provides an ideal model for examining the presence and constancy of the KoRV-A genome over the relatively short amount of time it has participated in the process of endogenization. Four museum koala skins from the late 19th century yielded suitable DNA for analysis by hybridization capture and next generation sequencing and provided a means of obtaining some answers to questions regarding early endogenization events [8]. Of the two DNA samples obtained from northeastern koalas skins both were KoRV-A positive whereas neither of the two koala skin samples from Southeastern Australia were KoRV-A positive. Of the 15 more recent museum skin DNA samples dating from the 20th century, 13 of the 14 northeastern koala DNA samples were KoRV-A positive. The sole southeastern koala sample was positive for KoRV-A [8]. By 2004, 98 of the 98 northeastern koalas tested were KoRV-A positive and eight of the 48 southeastern koalas samples were positive for KoRV-A by PCR performed on extracted genomic DNA [7]. These findings suggest that KoRV has spread across Northeast Australia, but there is limited spread among koalas inhabiting the southeast.

The 15 tested Northeastern koalas alive from 1870–1980 were positive for presumably endogenous KoRV-A provirus [8]. Whether KoRV-A could superinfect these animals remains an open question. However a study published in 2015 suggests that KoRV-A does not superinfect koalas harboring endogenized KoRV-A [9]. In that study, all 39 proviral loci identified in the sire and dam of a Queensland sire-dam-progeny triad of koalas were vertically transmitted to progeny. No new proviral loci were found in their progeny. The absence of new proviral integrants in the offspring argues against superinfection by endogenous KoRV [9]. 

The discovery of a heterogeneous population of KoRV envelope genes found in the genomic DNA and plasma of some koalas is similar to envelope variations found among feline leukemia virus (FeLV) isolates. This suggests the possibility that KoRVs may use mechanisms similar to FeLVs to overcome superinfection blocks and broaden host tropism [10]. In this paper we tested this hypothesis by using the mixed population of KoRV-A and KoRV-B viruses for infection to study the pseudotyping of KoRV-A using KoRV-B envelopes. We also investigated the heterogeneity of KoRV envelopes in koalas in U.S. zoos. Our findings suggest that more KoRV variants exist in koala population than was originally suggested in the first several years of KoRV research following the initial cloning of KoRV-A in 2000 [4,5,8,11]. These envelope variants can serve as candidates for recombination with KoRV-A to alter KoRV-A’s cellular tropism and overcome the interference block posed by endogenous KoRV-A.

## 2. Materials and Methods

### 2.1. Plasmids

pKoRVB-GFP was constructed by replacing GALV *env* in GALV-GZAP [12] with the KoRV-B envelope. All PCR amplicons of KoRV variants were cloned into pCI-neo expression vector.

### 2.2. Cells

The cells used in these studies include: 293T human embryonic kidney cells (ATCC CCL 11268), murine *Mus dunni* tail fibroblast MDTF cells [13], human HT1080 cells (ATCC CCL-121), bat lung cells (ATCC CCL 88), human HOS cells (ATCC CRL 1543), and rat XC cells (ATCC CCL 165). MDTF cells expressing the FeLV-C receptor members 1 and 2 were generously provided by Dr. C. Tailor (University of Toronto, Toronto, Canada). The generation of MDTF-PiT1 and MDTF-THTR1 to individually express human PiT1 and human THTR1 and HT1080 cells to express KoRV-A envelope protein were described in detail previously [14]. HT1080 cells infected with KoRV-B were generated in the same manner described for HT1080 cells infected with KoRV-A [14]. 293T-RT43.2GFP cells expressing a replication defective retroviral genome encoding GFP were established as previously described with >90% of the transduced 293T cells expressing GFP as determined by flow cytometry [14]. All cell lines were maintained in DMEM with high glucose, supplied with 10% FBS, 100 U of penicillin/mL, and 100 μg of streptomycin/mL. 

### 2.3. Transfection and Transduction

Transfection of 293T cells was carried out with ProFection Calcium Phosphate Transfection kit (Promega). Retroviral particles were produced by co-transfection of a pCI-neo plasmid encoding individual KoRV envelope (KoRV-A, KoRV-B, KoRV-E and KoRV-F), an MLV *gagpol*, and a retroviral genome encoding β-galactosidase (LacZ gene) as an indicator of transduction. Viral supernatants were then passed through a 0.45 µm syringe filter and stored at −80 °C. Transduction of target cells was carried out by seeding the cells at a density of 4 × 10^4^ cells/well in a 24-well plate for 24 h. The cells were then transduced with viral vectors pseudotyped with KoRV-A, KoRV-B, KoRV-E or KoRV-F envelopes in the presence of 10 µg/mL polybrene. Forty-eight hours post exposure to vectors, X-Gal (5-bromo-4-chloro-3-indolyl-β-D-galactopyranoside) staining was performed and blue colonies resulting from the expression of β-galactosidase were indicative of transduced cells.

### 2.4. Preparation of Koala Samples

The collection of koala blood and tissue samples, preparation of peripheral blood mononuclear cells (PBMCs) and plasma from blood samples, and purification of blood and tissue genomic DNA were carried out as previously described [14]. Stimulated PBMCs were co-cultured with 293T-RT43.2GFP cells for four to eight weeks and viral supernatants were collected, filtered through a 0.45 µm syringe and applied to the medium of murine MDTF cells expressing either the THTR1 or PiT1 receptor. The expression of GFP in KoRV-infected cells was verified by fluorescent microscopy. KoRV infection was confirmed by PCR amplification of genomic DNA prepared from MDTF-PiT1 or THTR1 cells infected by the various KoRV subgroups.

### 2.5. PCR and RT-PCR

**Figure 1:**
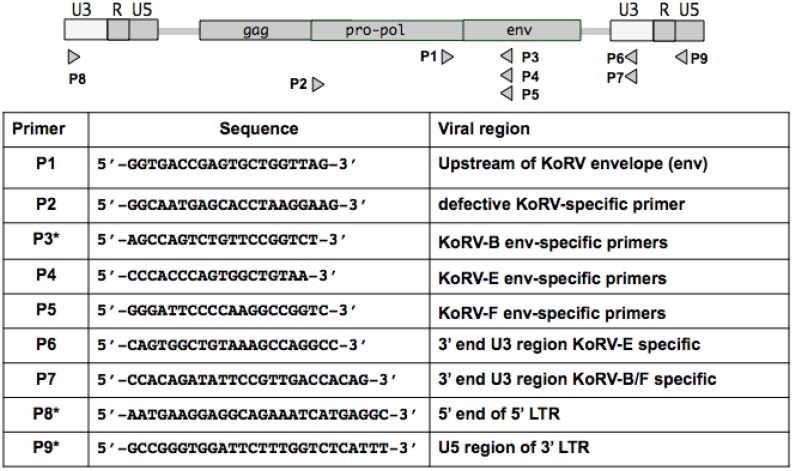
Schematic representation of the KoRV proviral genome showing positions of primer pairs used to clone KoRV envelope variants and defective KoRV envelopes. Diagram not to scale. *Primers used in previous studies [3,14].

Primer sequences used for amplification of KoRV variants are listed in Figure 1. The defective KoRV-B provirus was PCR-amplified using LA Taq DNA Polymerase (Takara) according to the instruction with the two primer sets P8/P3 and P2/P9 to generate two overlapping halves of the defective KoRV-B genome PCR. Takara PrimeSTAR HS DNA Polymerase was used to PCR amplify the full-length KoRV-E and KoRV-F envelope genes with primer pair P1/P6 for KoRV-E and P1/P7 for KoRV-F.

The RT-PCR assay to detect KoRV variants in koala plasma was performed using envelope specific primers of KoRV-E (P4) or KoRV-F (P5) paired with P1 primer complementary to the sequences upstream of the envelope gene of all KoRV variants. The isolation of viral RNA using QIAamp Viral RNA Mini Kit (Qiagen, Valencia, CA, USA) and removal of contaminating DNA was described previously [14]. A SuperScript First-Strand synthesis kit (Invitrogen, Carlsbad, CA, USA) was also used for first strand cDNA synthesis of RNA prepared from plasma. The reverse transcriptase-negative and RNA template-negative controls were performed in parallel for each of the analyzed sample.

## 3. Results

### 3.1. KoRV-B Envelope Can Pseudotype Infectious KoRV-A Viral Particles

**Figure 2:**
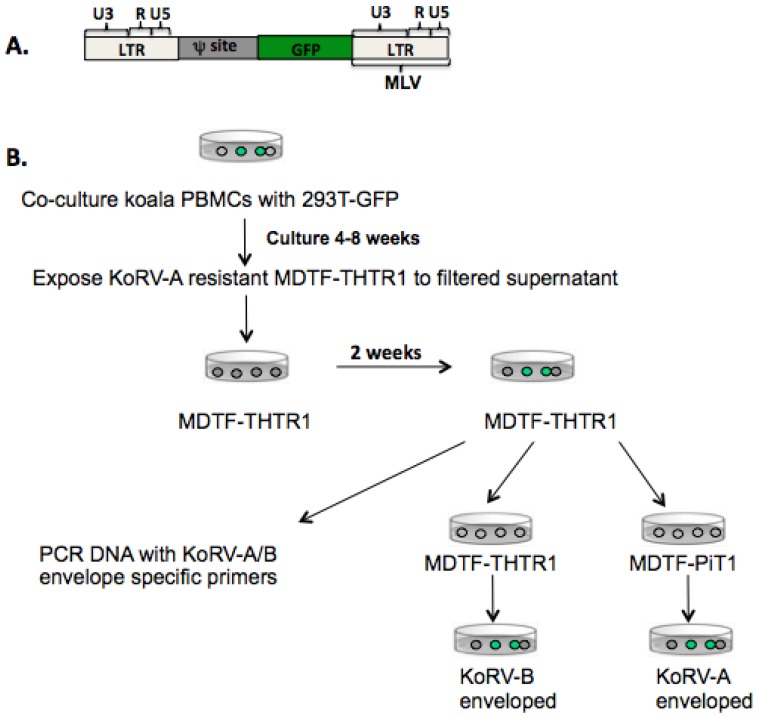
(**A**) Genomic organization of the defective GFP expressing retroviral vector. The long terminal repeats (LTRs) including U3, R, and U5 regions are derived from Moloney murine leukemia virus (Mo-MLV). The defective GFP genome is integrated into the genome of 293T-GFP cells and can be rescued after co-culture with koala PBMCs harboring infectious KoRVs; (**B**) Experimental design used to determine whether KoRV-B envelopes can pseudotype the KoRV-A genome. Green-filled circles indicate GFP expressing cells. Infectious KoRV-B enveloped viruses obtained by co-culturing 293T-GFP with koala PBMCs were used to infect KoRV-A-resistant MDTF-THTR1 cells. Wildtype and pseudotyped viruses in the supernatant of infected MDTF-THTR1 cells were subsequently used to infect MDTF-PiT1 and MDTF-THTR1 cells.

PBMCs collected from all tested koalas in U.S. zoos produce KoRV-A. [14]. We, and others, have found that all KoRV-B infected animals also harbor KoRV-A [14,15,16]. Previously we demonstrated that we could perform a marker rescue assay with KoRV-B infected koala PBMCs co-cultured with 293T cells that express a defective MLV genome encoding GFP (designated RT43.2GFP, Figure 2A) to allow the recovery of either KoRV-A and KoRV-B enveloped GFP vector particles as well as replication competent KoRV-A and KoRV-B [14]. Murine MDTF cells are resistant to KoRV-A and KoRV-B enveloped particles. Expression of the KoRV-A receptor, PiT1, or the KoRV-B receptor, THTR1, confers susceptibility to KoRV-A or KoRV-B, respectively. We employed MDTF/THTR1 and MDTF/PiT1 to determine whether KoRV-B can pseudotype KoRV-A genomes using a protocol schematically shown in Figure 2B. PBMCs obtained from a koala infected with KoRV-A and KoRV-B were co-cultured for four to eight weeks with 293T-RT43.2GFP cells. MDTF/THTR1 exposed to filtered supernatant from these co-cultured cells were passaged for two weeks at which time genomic DNA was prepared and PCR amplified fragments were obtained using KoRV-A or KoRV-B specific primers. The presence of KoRV-A specific proviral sequences was consistent with KoRV-B pseudotyping KoRV-A. Furthermore, supernatant harvested from these cells contained both KoRV-A and KoRV-B enveloped particles as evident by the expression of GFP in both the MDTF/THTR1 and MDTF/PiT1 48 h post-exposure to vectors (Figure 2B). Thus, KoRV-B can pseudotype KoRV-A.

### 3.2. Detection of Two Additional KoRV Envelope Variants by PCR Amplication

**Figure 3:**
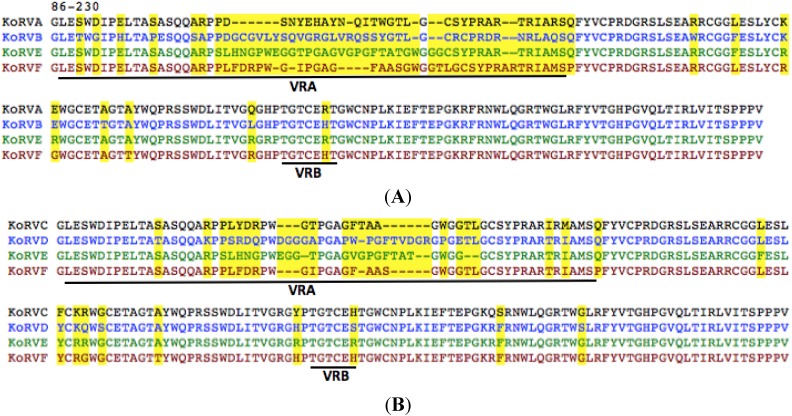
(**A**) KoRV-A, B, E, and F envelope proteins differ mainly in the VRA region. Alignment of the amino terminus of KoRV-A,-B,-E and -F (corresponding to residues 86–230 of KoRV-A) envelope proteins. Residues that differ among this envelope segment are highlighted in yellow; (**B**) Predicted KoRV-F residues comprising the amino terminal region of the envelope protein aligned to the corresponding regions of KoRV-C, D, and E and F residues (residue numbers correspond to KoRV-A envelope residues 86–230). The putative VRA and VRB regions are underlined with residues that differ among the four isolates highlighted in yellow.

We have previously reported the isolation and sequencing of a novel exogenous KoRV isolate designated KoRV-B genome [14]. We used similar PCR-based methodologies to identify two additional KoRV envelope genes in genomic DNA prepared from koala PBMCs and as envelope transcripts in the plasma of several koalas. The residues encoded by these two envelope ORFs, now designated KoRV-E and KoRV-F are shown in Figure 3A. KoRV-E and KoRV-F differ from both KoRV-A and KoRV-B in the VRA regions of their envelope RBD. As shown in Figure 3B, KoRV-E represents a unique variant containing a VRA region that differs from the previously characterized KoRV envelope variants obtained from koalas in Japanese zoos [17]. To determine what receptors bind KoRV-E and KoRV-F, we cloned the envelope ORFs of KoRV-E and F in an expression vector and constructed vectors pseudotyped with either KoRV-E or KoRV-F. KoRV-E vectors, like vectors bearing KoRV-A or KoRV-F envelopes were able to transduce human HT1080 and 293T, but not murine MDTF cells (Supplementary Table S1). KoRV-E also infects HT1080 cells productively infected with GALV (GALV-GZAP, [12]), HT1080 cells infected with KoRV-A that expresses GFP (KoRVA-GFP, [14]) in a manner analogous to GALV-GZAP and HT1080 cells infected with KoRV-B (KoRVB-GFP). None of the three HT1080 cells expressing the GALV, KoRV-A or KoRV-B envelope blocked infection by KoRV-E, suggesting that KoRV-E does not use the receptor employed by GALV and KoRV-A or KoRV-B.

Next, we expressed a panel of known human transporters used by other gammaretroviruses in KoRV-E resistant cells, such as MDTF. Their expression was verified by transduction of the appropriate enveloped vectors. We thereby determined that the receptor for murine amphotropic retroviruses, the inorganic phosphate transporter 2, PiT2 or SLC20A2 [18,19], the porcine endogenous virus A (the riboflavin transporter SLC52A1 [20]), the sodium-dependent neutral amino acid transporter type 1 (SLC1A5) the receptor for baboon endogenous virus, feline endogenous virus RD114 and other gammaretroviruses [21,22], the FeLV subgroup C receptor-the heme transporter [23], or the receptors for xenotropic and polytropic MLVs [24] do not facilitate entry of KoRV-E enveloped vectors when expressed in the appropriate target cells.

### 3.3. The KoRV-F Env ORF Is Closely Related to Previously Characterized KoRV Variants

**Figure 4:**
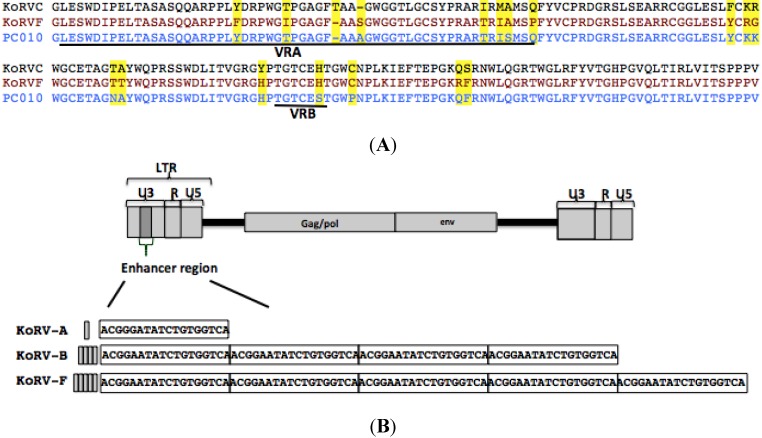
(**A**) The VRA region of KoRV-F is related to VRA regions of KoRV-C and PC010 KoRV envelope [16]. Envelope residues corresponding to KoRV-A envelope residue numbers 86–230 are aligned. VRA and VRB regions are underlined. Residues that differ among KoRV-E, F and PC010 are highlighted in yellow; (**B**) Schematic representation of the sites where the 18 nucleotide repeats are positioned within the U3 enhancer regions (dark grey) of KoRV-B and KoRV-F. The grey bar represents the tandem repeat of the 18-nucleotide insertion, with KoRV-A containing a single copy, KoRV-B four copies and KoRV-F five copies. The 18 nucleotides are boxed.

KoRV-F contains a VRA region that differs from KoRV-A, B, or E. However alignment of KoRV-F to the previously identified KoRV-C envelope from Japan [17] and the env PC010 ORFs isolated from wild type koalas from Australia using transcriptomic analysis [15] showed that KoRV-F shares 95% residue identity with KoRV-C and 96% residue identity with PC010. Most importantly, the residue difference between KoRV-F and KoRV-A, B, or E is concentrated in the variable region A (VRA) of the envelope, however, as shown in Figure 4A, KoRV-F shares a similar VRA region to KoRV-C and PC010. Residue differences among these isolates is not exclusively concentrated in the VRA region but are variably distributed throughout different envelope regions. KoRV-F envelope pseudotyped MLV vectors failed to infect any cells assessed including human cells and bat cells (Supplementary Table S1). KoRV-F specific sequences were detected in 293T cells co-cultured with the PBMCs of several koalas (#3,4,5,6,and 8) in U.S. zoos, which suggests that a KoRV-F viral genome was pseudotyped by a KoRV envelope that can infect 293T cells. Using a 3' primer complementary to the U5 region of KoRV-B LTR (P7 in Figure 1), we were able to sequence the U3 region of KoRV-F downstream of the KoRV-F ORF envelope. The KoRV-F U3 contains five repeats of the enhancer segment compared to the four repeats found in the KoRV-B and the single repeat present in KoRV-A U3 [14] and Figure 4.

### 3.4. KoRV-E and KoRV-F Are Not Transmitted as Endogenous Retroviral Elements

**Figure 5:**
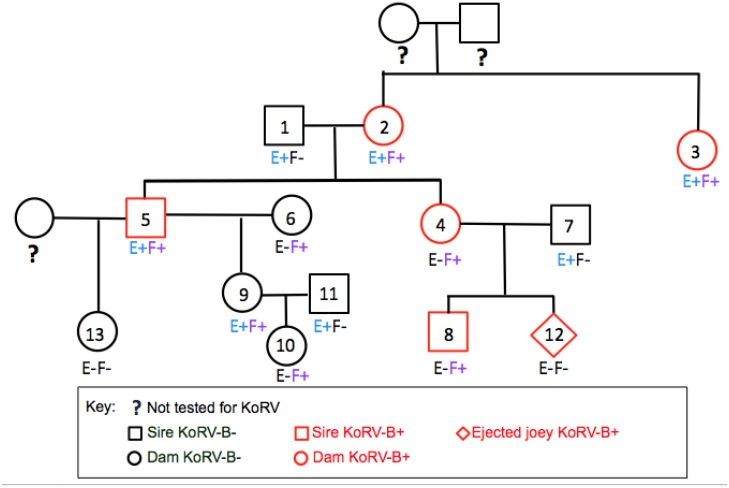
KoRV-E and KoRV-F are not vertically transmitted. All koalas are KoRV-A positive. Male and female koalas are denoted as square and circle symbols, respectively, in the family tree. KoRV-E positive koalas are denoted as E+ and KoRV-F positive koalas as F+.

In previous studies, in which koalas from the LA zoo were assessed, we discovered that KoRV-B was transmitted from dams but not sire to offspring [14]. A pedigree analysis was undertaken for KoRV-E and KoRV-F infection similar to the lineage analysis we previously published for KoRV-B [14]. As shown in Figure 5, koalas that are infected with KoRV-B are not unvaryingly KoRV-E or KoRV-F positive. Furthermore KoRV-E positive koalas did not uniformly give rise to KoRV-E positive offspring. Both koalas #1 and #2 are KoRV-E positive, but their offspring #5 is KoRV-E positive, whereas offspring #4 is KoRV-E negative. Similarly koalas #9 and #11 are KoRV-E positive, but their offspring, #10, is not KoRV-E positive. The #10 offspring of KoRV-F positive koalas #5 and #6 are similarly F positive, but the #13 offspring of KoRV-F positive #5 is KoRV-F negative. These results suggest that KoRV-E and KoRV-F are not invariably vertically transmitted from parents to offspring.

### 3.5. Defective KoRV-B, -E and -F Were Detected in Koalas Containing Intact KoRV-B Virus or KoRV-E and -F Envelopes

Using KoRV-B specific primers, we also isolated a KoRV-B-like genome, which has a full LTR region similar to the originally isolated KoRV-B [14], partial pol sequences, and sequences highly related to KoRV-B env. However, this KoRV-B variant contains ACTG residues in place of ATG starting codon in the originally characterized KoRV-B envelope ORF. This variant contains partial gag sequences that would be predicted not to encode gag gene products (Supplementary Figure S1). The defective KoRV-B env was detected in all KoRV-B-positive koalas. We detected both KoRV-B and defective KoRV-B in koala #19 from Australia (Supplementary Table S2). Furthermore, we also detected defective KoRV-B sequences in a koala also born in Australia that was KoRV-B negative. A defective KoRV-B was detected in a KoRV-B-negative koala recently imported from Australia (koalas #16 and #18 in Supplementary Table S2). Defective envelope sequences that result from the substitution of ACTG for the KoRV-B envelope ATG were also detected in KoRV-F envelopes (Supplementary Figure S2). The defective envelopes share 99% identity with their functional envelope counterparts.

## 4. Discussion

The genomes of KoRV-A and KoRV-B are highly conserved with variability occurring mainly in the envelope gene and in the U3 or viral promoter region of the LTR. The sequenced regions of KoRV-F genome also show variability in the U3 and receptor-binding domain of the envelope when compared to KoRV-A and KoRV-B (Figure 3 and Figure 4). The single 18-nucleotide enhancer region present in KoRV-A is present as five tandem repeats in KoRV-F (Figure 4B). This 18-nucleotide enhancer region contains several transcription factor binding sites include those for transcription factors TCF3, GATA-1, MafG, Myb, and Spi-B [14]. Enhancer-mediated recruitment of transcription factors are integral to the regulation of host genes, some with oncogenic potential [10].

**Figure 6:**
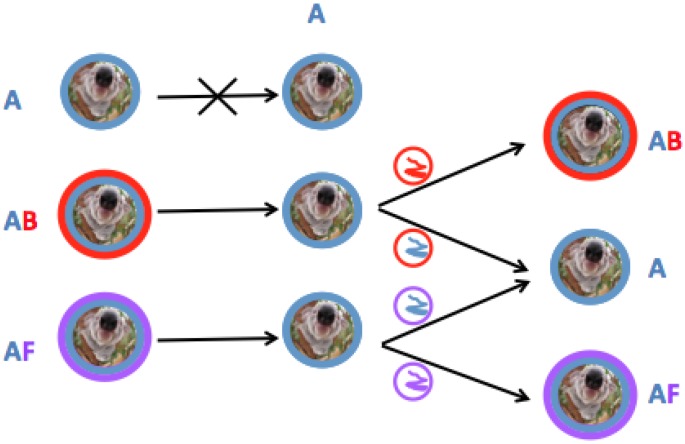
Mechanism of KoRV enveloped retroviral transmission in koalas infected with KoRV-A (represented as koalas within blue circles), KoRV-B (red encircled koalas) and/or KoRV-F (koalas within purple circles). The small circles represent KoRV viral particles composed of KoRV-A, B, or F envelopes (blue, red or purple, respectively) and the folded lines within the circles represent the KoRV-A, KoRV-B or KoRV-F genomes (red, blue or purple lines, respectively). KoRV-A positive animals are resistant to superinfection by KoRV-A (top most figure) but the KoRV-A genome (blue) can be transmitted in particles bearing KoRV-B (red) or KoRV-F (purple) envelopes.

KoRV-A is a newly endogenized retrovirus that has been resident in the koala genome for several decades to a maximum age of approximately 22,200–49,900 years ago [9,25]. To date, there exists no evidence of KoRV-A horizontal transmission as an infectious virus [9]. KoRV-B and KoRV-F have only been isolated from koalas infected with KoRV-A [14,15]. Thus KoRV-B and KoRV-F may provide a means of mobilizing endogenous KoRV-A by circumventing the superinfection interference block present in koala in which KoRV-A exists as an endogenous virus. Indeed it has been demonstrated that KoRV transcript levels in plasma are significantly increased in animals suffering from neoplasias such as lymphoma when compared to KoRV-A positive healthy animals [26]. Taking into account that the primers used in these studies amplify a region of the polymerase gene conserved between KoRV-A and other KoRV variants the observed increased viral RNA levels could be attributable to KoRV-B or KoRV-F (Figure 6). The genome of KoRV-F has not been sequenced in its entirety, therefore, it is presently unknown whether KoRV-F represents a defective or full length replication competent genome.

The absence of KoRV-E or KoRV-F in the offspring of KoRV-E or KoRV-F positive parents suggests KoRV-E and KoRV-F are not invariably vertically transmitted (Figure 5). KoRV-A is a young endogenous virus and KoRV loci do not appear to be fixed in the population. This was dramatically illustrated when it was shown that all 38 KoRV-A loci not shared between a dam or sire were vertically transmitted to the offspring [9]. This report demonstrated that loci are transmitted efficiently to offspring a situation not observed with either KoRV-B [14] or KoRV-F (Figure 5).

## 5. Conclusions

In conclusion, in contrast to the belief held for more than a decade that KoRV-A was an invariant species of virus we have now determined that there is considerable variation in the envelope regions as well as the LTRs of KoRVs. The consequence of this variation is a genetically diverse virus population that will most likely be continuously shaped by selective pressures. The nature of KoRV-B and KoRV-E and KoRV-F variants, their relationship to malignant disease, the cellular factors that contribute to disease and the selective mechanisms leading to their predominance are questions that need to be addressed to resolve KoRV-mediated pathogenesis.

## Supplementary Files

Supplementary File 1
