# Supplementary material for: Genetic Diversity of Koala Retroviral Envelopes
Source: Viruses. 2015 Mar 17;7(3):1258–70. doi: 10.3390/v7031258 (PMC4379569; doi:10.3390/v7031258)
Supplement: Supplementary File 1 [file viruses-07-01258-s001.pdf]

Suppl. Figure 2. Nucleotide sequence alignment of envelope genes of KoRV-F and env-like region of defective KoRV-F (Def-KF). Identical sequences are denoted with * below the alignment. Nucleotides that differ are highlighted in green.

```
KoRV-F   A-TGCTTCTCATCTCAAACCCGCGCCACCTGGGGCACCCGATGAGTCCTGGGAACTGGAAAAGACTGATCATCCTCTTAAGTTGCGTGTTCGGCGGCGCCGAAATGAATCAACAGCATAAAAGACTGATCATCCTCTTA   138
Def-KF   ACTGCTTCTCATCTCAAACCCGCGCCACCTGGGGCACCCGATGAGTCCTGGGAACTGGAAAAGACTGATCATCCTCTTAAGTTGCGTGTTCGGCGGCGCCGAAATGAATCAACAGCATAAAAGACTGATCATCCTCTTA   139
         * *********************************************************************************************************************************

KoRV-F   AGTTGCGTGTTCGGCGGCGCCGAAATGAATCAACAGCATAACAACCCTCACCAACCCATGACTCTCACCTGGCAGGTACTGTCCCAGACGGGAAGTGTCGTTTGGGAAAAGAAAGCAGTCGAGCCACCCTGGACGTGGT   277
Def-KF   AGTTGCGTGTTCGGCGGCGCCGAAATGAATCAACAGCATAACAACCCTCACCAACCCATGACTCTCACCTGGCAGGTACTGTCCCAGACGGGAAGTGTCGTTTGGGAAAAGAAAGCAGTCGAGCCACCCTGGACGTGGT   278
         *******************************************************************************************************************************************

KoRV-F   GGCCCTCTCTTGAGCCTGATGTGTGCGCACTGGTAGCCGGTCTCGAGTCCTGGGACATCCCGGAACTCACTGCATCGGCCTCTCAACAGGCCAGGCCACCTCTGTTTGACCGGCCTTGGGGAATCCCGGGGGCTGGCTT   416
Def-KF   GGCCCTCTCTTGAGCCTGATGTGTGCGCACTGGTAGCCGGTCTCGAGTCCTGGGACATCCCGGAACTCACTGCATCGGCCTCTCAACAGGCCAGGCCACCTCTGTTTGACCGGCCTTGGGGAATCCCGGGGGCTGGCTT   417
         *******************************************************************************************************************************************

KoRV-F   TGCAGCCTCCGGATGGGGGGGAACCCTAGGGTGTAGCTATCCCCGGGCCAGAACAAGAATTGCTATGTCCCCGTTTTACGTGTGCCCCCGGGATGGCCGTTCCTTGTCCGAGGCCCGGAGGTGCGGAGGACTCGAATCT   555
Def-KF   TGCAGCCTCCGGAtGGGGGGGAACCCTAGGGTGTAGCTATCCCCGGGCCAGAACAAGAATTGCTATGTCCCCGTTTTACGTGTGCCCCCGGGATGGCCGTTCCTTGTCCGAGGCCCGGAGGTGCGGAGGACTCGAATCT   556
         *******************************************************************************************************************************************

KoRV-F   TTATACTGTAGAGGGTGGGGTTGTGAGACCGCGGGAACCACTTACTGGCAACCCCGGTCCTCGTGGGACCTTATAACTGTAGGCCGGGGCCACCCTACGGGGACATGCGAACACACCGGTTGGTGCAACCCACTCAAGA   694
Def-KF   TTATACTGTAGAGGGTGGGGTTGTGAGACCGCGGGAACCACTTACTGGCAACCCCGGTCCTCGTGGGACCTTATAACTGTAGGCCGGGGCCACCCTACGGGGACATGCGAACACACCGGTTGGTGCAACCCACTCAAGA   695
         *******************************************************************************************************************************************

KoRV-F   TAGAGTTCACCGAGCCTGGCAAACGGTTCCGAAACTGGCTACAGGGGCGAACCTGGGGTCTAAGGTTCTATGTGACTGGACACCCAGGCGTGCAGTTGACCATACGACTAGTGATCACAAGTCCCCCACCGGTAGTAGT   833
Def-KF   TAGAGTTCACCGAGCCTGGCAAACGGTTCCGAAACTGGCTACAGGGGCGAACCTGGGGTCTAAGGTTCTATGTGACTGGACACCCAGGCGTGCAGTTGACCATACGACTAGTGATCACAAGTCCCCCACCGGTAGTAGT   834
         *******************************************************************************************************************************************

KoRV-F   GGGCCCCGATCCTGTCCTCGCAGGACAAGGACCTCCCAGAAAAATTCCCTTCCTCCCCAGAGTGCCGGTTCCTACCCTATCACCCCCAGCGTCCCCTATCCCCACGGTACAGGCTAGCCCCCCGGCCCCTAGTACTCCA   837
Def-KF   GGGCCCCGATCCTGTCCTCGCAGGACAAGGACCTCCCAGAAAAATTCCCTTCCTCCCCAGAGTGCCGGTTCCTACCCTATCACCCCCAGCGTCCCCTATCCCCACGGTACAGGCTAGCCCCCCGGCCCCTAGTACTCCA   838
         *******************************************************************************************************************************************

KoRV-F   TCTCCCACCACGGGCGACCGGCTCTTTGGCCTCGTACAAGGGGCCTTTCTGGCCTTGAATGCCACCAACCCGGAGGCCACGGAGTCTTGCTGGCTCTGTCTAGCCCTCGGCCCTCCTTATTACGAAGGAATCGCAACTC   972
Def-KF   TCTCCCACCACGGGCGACCGGCTCTTTGGCCTCGTACAAGGGGCCTTTCTGGCCTTGAATGCCACCAACCCGGAGGCCACGGAGTCTTGCTGGCTCTGTCTAGCCCTCGGCCCTCCTTATTACGAAGGAATCGCAACTC   973
         *******************************************************************************************************************************************

KoRV-F   CAGGGCAAGTCACTTATGCCTCCACAGATTCCCAATGTCACTGGGGAGGAAAAGGGAAGCTCACCCTCACTGAAGTCTCTGGACTCGGGTTATGTATAGGGAAAGTACCTCCTACTCACCAACATCTCTGCAACCTGAC   1111
Def-KF   CAGGGCAAGTCACTTATGCCTCCACAGATTCCCAATGTCGCTGGGGAGGAAAAGGGAAGCTCACCCTCACTGAAGTCTCTGGACTCGGGTTATGTATAGGGAAAGTACCTCCTACTCACCAACATCTCTGCAACCTGAC   1112
         *************************************** ***************************************************************************************************

KoRV-F   TATACCCTTAAACGCCTCACATACTCACAAGTACCTCCTCCCCTCCAACCACAGCTGGTGGGCTTGTAATTCTGGCCTCACCCCCTGCCTCTCTACATCAGTCTTCAACCAGTCTAACGATTTCTGTATCCAGATCCAG   1197
Def-KF   TATACCCTTAAACGCCTCACATACTCACAAGTACCTCCTCCCCTCCGACCACAGCTGGTGGGCTTGTAATTCCGGCCTCACCCCCTGCCTCTCTACATCAGTCTTCAACCAGTCTAACGATTTCTGTATCCAGATCCAG   1198
         ********************************************** ************************* *****************************************************************

KoRV-F   CTTGTCCCTCGCATCTACTATCACCCAGACGGTACCTTGCTACAGGCCTATGAGTCCCCCCACCCTAGAAACAAGAGAGAGCCTGTCTCACTCACCCTGGCTGTCCTTCTCGGATTAGGGGTCGCAGCAGGTATAGGTA   1250
Def-KF   CTTGTCCCTCGCATCTACTATCACCCAGACGGTACCTTGCTACAGGCCTATGAGTCCCCCCACCCTAGAAACAAGAGAGAGCCTGTCTCACTCACCCTGGCTGTCCTTCTCGGATTAGGGGTCGCAGCAGGTATAGGTA   1251
         *******************************************************************************************************************************************

KoRV-F   CCGGCTCGACCGCCCTAATAAAAGGGCCCATCAAGACCTCCAACAAGGTTTGACTAGCCTCCAAATTGCCATGGATACAGACCTTAGAGCCCTTCAAGACTCCGTAAGTCAACTAGAGAATTCCTTAACCTCCCTTTCT   1389
Def-KF   CCGGCTCGACCGCCCTAATAAAAGGGCCCATCAAGACCTCCAACAAGGTTTGACTAGCCTCCAAATTGCCATGGATACAGACCTTAGAGCCCTTCAAGACTCCGTAAGTCAACTAGAGAATTCCTTAACCTCCCTTTCT   1390
         *******************************************************************************************************************************************

KoRV-F   GAAGTAGTGCTCGAATAGGAGAGGCCTTGATCTGCTATTTTTGAAGGAAGGGGGCCTTTGTGCAGCCCTAAAAGAGGAGTGCTGTTTCTATGTTGACCACTCAGGCCCGGTGCGAGACTCCATGAGGAAACTCAAGGAA   1528
Def-KF   GAAGTAGTGCTCGAATAGGAGAGGCCTTGATCTGCTATTTTTGAAGGAAGGGGGCCTTTGTGCAGCCCTAAAAGAGGAGTGCTGTTTCTATGTTGACCACTCAGGCCCGGTGCGAGACTCCATGAGGAAACTCAAGGAA   1529
         *******************************************************************************************************************************************

KoRV-F   AGGCTAGATAAGAGGCAGTTAGAGCACCAAAAGAATTTAAGTTGGTACGAGGGATGGTTCAACCGTTCCCCCTGGCTTACTACTTTACTGTCTGCCCTTGCTGGTCCCTTGCTACTCCTCCTTCTGTTACTCACCCTCG   1667
Def-KF   AGGCTAGATAAGAGGCAGTTAGAGCACCAAAAGAATTTAAGTTGGTACGAGGGATGGTTCAACCGTTCCCCCTGGCTTACTACTTTACTGTCTGCCCTTGCTGGTCCCTTGCTACTCCTCCTTCTGTTACTCACCCTCG   1668
         *******************************************************************************************************************************************

KoRV-F   GGCCTTGTGTCATCAATAAGTTAGTGCAATTCGTCAATGATAGGGTTAGTGCAGTAAGAATCCTGTTCCTCAGACACAAGTACCAGACCCTAGACAACGAGGATAACCTTTAA   1780
Def-KF   GGCCTTGTGTCATCAATAAGTTAGTGCAATTCGTCAATGATAGGGTTAGTGCAGTAAGAATCCTGTTCCTCAGACACAAGTACCAGACCCTAGACAACGAGGATAACCTTTAA   1780
         *****************************************************************************************************************
```

Suppl. Figure 1. Alignment of KoRV-B with defective KoRV-B (Def-B) sequences. Def-B sequences were obtained from two overlapping PCR amplicons obtained with primer pairs P8/P3 and P2/P7. The sequences of these primers are underlined in the text. Identical nucleotides are denoted with a short bar between the nucleotide sequences. Dashes indicate gaps between sequences. Square brackets are used to indicate major sections of the KoRV-B genome including 5' LTR, gag, pro-pol, env [14]. Env sequences are also colored blue for KoRV-B, and nucleotides that differ between the regions of KoRV-B env and Def-B env-like sequences are highlighted in green.

```
          5′ LTR
KoRV-B  [AATGAAGGAGGCAGAAATCATGAGGCAGAAATCATTCCGTGAAGTATGGAAACTACCCGGAGGGCCCCAGGGTTAGGGACAAGCGCAGCCAGGCACAGTAAAAGGTCAGAGCAAGAAAAA  120
        ||||||||||||||||||||||||||||||||||||||||||||||||||||||||||||||||||||||||||||||||||||||||||||||||||||||||||||||||||||||||
Def-B   AATGAAGGAGGCAGAAATCATGAGGCAGAAATCATTCCGTGAAGTATGGAAACTACCCGGAGGGCCCCAGGGTTAGGGACAAGCGCAGCCAGGCACAGTAAAAGGTCAGAGCAAGAAAAA  120
        ‾‾‾‾‾‾‾‾‾‾‾‾‾‾‾‾‾‾‾‾‾‾‾‾‾‾
                 primer:P8

KoRV-B  CAAGGAAGGTTTGGAGTGCCAAACGGAATATCTGTGGTCAACGGAATATCTGTGGTCAACGGAATATCTGTGGTCAACGGAATATCTGTGGTCATGCACCTGGGTCCCCACCCCGGACTT  240
        |||||||||||||||||||||||||||||||||||||||||||||||||||||||||||||||||||||||||||||||||||||| |||||| ||||||||| ||||||||||||||||
Def-B   CAAGGAAGGTTTGGAGTGCCAAACGGAATATCTGTGGTCAACGGAATATCTGTGGTCAACGGAATATCTGTGGTCAACGGAATATCAGTGGTCGTGCACCTGAGTCCCCACCCCGGACTT  240

KoRV-B  ATGCAAACAATTCCCAGAAATAGCTGAGCTCATAACAGTTTCTAGGGTGCCCCTCAGCAGTTTCTAGAACCCTCTCGTGACCGGAGTTTTTATTCAAACTAACCAATGATCTTGCTCCTC  360
        ||||||||||||||||||||||||||||||||||||||||||||||||||||||||||||||||||||||||||||||||||||||||||||||||||||||||||||||||||||||||
Def-B   ATGCAAACAATTCCCAGAAATAGCTGAGCTCATAACAGTTTCTAGGGTGCCCCTCAGCAGTTTCTAGAACCCTCTCGTGACCGGAGTTTTTATTCAAACTAACCAATGATCTTGCTCCTC  360

KoRV-B  GCTTCTGTACCCGCGCTTTTTTGCTATAAAATGAGACCAAAGAATCCACCCGGCGCGCCAGTCCCTCTAGGTGACTGAGTCGCCCGAGTACTCGTAAGTTCAATAAACCTCTTGCTATTT  480
        |||||||||||||||||||||||||||||||||||||||||||||||||||||||||||||||||||||||||||||||||||||||||||||| |||||| ||||||||||||||||||
Def-B   GCTTCTGTACCCGCGCTTTTTTGCTATAAAATGAGACCAAAGAATCCACCCGGCGCGCCAGTCCCTCTAGGTGACTGAGTCGCCCGAGTACTCGCAAGTTC-ATAAACCTCTTGCTATTT  479

                                                               5′ LTR
KoRV-B  GCATCCGGAGTTGTGTTCGCGTTGATCCTGGGAGGGTTTCTCAAGGTC]GGAGGACTACCCGAAATTCGGGGTCCAACATTTGGGGGCTCGTCCGGGATCTGAGATTCCCACCCAAGGACC  600
        ||||||||||||||||||| ||||||||||||||||||||||||||||||||||||||||||||||||||||||||||||||||||||||||||||||||||||||||||||||||||||
Def-B   GCATCCGGAGTTGTGTTCGTGTTGATCCTGGGAGGGTTTCTCAAGGTCGGAGGACTACCCGAAATTCGGGGTCCAACATTTGGGGGCTCGTCCGGGATCTGAGATTCCCACCCAAGGACC  599

KoRV-B  GCCGAACCACCGACGGGAGGTAAGCTGGCCAGCGATCGCTCTATGTTCTCTCTGTGTCTGACTCCGAAAACTCTGACTGTCTATTCGGTGTGCGCGCATTTTTGGTTTCAGTTTATTCCG  720
         |||| ||||||||||||||||||||||||||||||||||||||||||||||||||||||||||    |||||||||||||||||||||||||||||| ||||||| |||||||||||||
Def-B   ACCGAGCCACCGACGGGAGGTAAGCTGGCCAGCGATCGCTCTATGTTCTCTCTGTGTCTGACTC----AACTCTGACTGTCTATTCGGTGTGCGCGCAATTTTGGTATCAGTTTATTCCG  715

KoRV-B  GGTTAATCGATCTAAGATCGAGGCGTGAGTAGCGGACAGACGTGTTCGGGGGCTCACCGCCCGGCAATCCTGGGAGACGTCCCAGGATCAGGGGAGGACCAGGGACGCCTGGTGGA----  836
        ||||||||||||| ||||||||| ||||||||||||||||||||||||||||||||| ||||||||||||||||||||||||||||||| |||| |||||||||||||||||||||
Def-B   GGTTAATCGATCTGAGATCGAGGTGTGAGTAGCGGACAGACGTGTTCGGGGGCTCACTGCCCGGCAATCCTGGGAGACGTCCCAGGATC-GGGG-GGACCAGGGACGCCTGGTGGACCCT  833

KoRV-B  ------------------------------------------------------------------------------------------------------------------------  836

Def-B   GCGGGTGAGGGGTAAAACCCTTTCGGTATCGGTAGGCTCTTTTTTTCTTGTCTTTTTCGTGTGTGTCAATGCCGGCGTGTTGTCTGGGATGCAAAAGGAATGTTCCACATTCAGTAGTGT  953

KoRV-B  ------------------------------------------------------------------------------------------------------------------------  836

Def-B   TGAACTCTGTGCCTAAGGTTTTGAATTACTTTTATTCTTCACTGTAATTGTCGACATGGACTGGGGCGGGGGGGGAGTCATGAAAAGTGAATATTGACGCAACAGAAATGCATGAATTTC  1073
```

```
KoRV-B  -----------------------------------------------------TCCC--ACGGCAAGGGATAATTCTCTTCTGATCTCACAGTTGCACTGTCTTGAGAGACATACCCTAC  901
                                                              |    |  |||     |  || | || |  || ||| ||   |  ||     | |  |      |
Def-B   AATATACTTTTATCTTTAGATGACACCATTTGAAGAAACTGGTATTATGAAGAACATAAAT-GCACT--GTTTTTATTTTATTTTCCCACTGTCTTAAAGTGGAAGGTGTTAGGTTAAAT  1190

                                                                                                                               gag
KoRV-B  CTTCTGACTCTTTTTCCTGTCTTTTTAATATACGTCTACGCCGCCATATAATTTTTTTCCTTTTTATTTCAATAATAATCATTATCTGGGTCGTAATTCCACTCTTCGGGACCCCCAGAA  1021
                    ||    |  |    |    ||    |  |     |    ||   ||  |  |    |       |   |      |  |  |  ||  |         |
Def-B   ACAAAATACAAATTGATAGGTTGGCCACATAACAAGGAAAATGTGAATGTTTAAAATTGGCTTAAAGATTTCCATGTGCAGTGTACATTTGTGACAAGCTTCTAATTCCACAGAACCCTC  1310

KoRV-B  TGGGACAGGGTGAGTCGACCCCTCTCTCTCTTACACTAGACCACTGGAAAGACGTGAAGACAAGGGCTCACAATCTTTCCGTGGAGATAAGAAAGGGAAAGTGGCAAACCTTCTGTTCCT  1141
         ||   |       ||   | |||| | ||           ||||| || | ||||| | ||||||||||||||||||| |||||||    || || ||| ||||||||| |||||||||
Def-B   AGGTTTAATCGATCTCTCTCTCTCTTTATCCACACTGGTTTCACTGAAACGTCGTGAGGTCAAGGGCTCACAATCTTTCAGTGGAGACGCAAAGGGAAAAATGGCAAACCCTCTGTTCCT  1430

KoRV-B  CCGAGTGGCCCACGTTCGAAGTGGGATGGCCACCGGAGGGGAC-TTTTAATCCTTCTATTATTTCTGCA-GT-CAAAAGGATTGTCTTCCAGGAGACTGGAGGACACCCGGACCAGGTTC  1258
        |||||||||||||||| || |||||||| |||| ||||||||  |||||||||||||||||||||| ||     |   |   | |     ||       ||   |      |
Def-B   CCGAGTGGCCCACGTTTGAGGTGGGATGTCCACAGGAGGGGAATTTTTAATCCTTCTATTATTTCTACAGTCAAAGCGGTTGTCTTCCGGAGACTGGAAGACACCCGGACTAGATTTCCT  1550

KoRV-B  CCTACATCATAGTTTGGCAGGACCTCTCCAACAGCCCCCCCCCATGGGTGCCACCCTTAGCCAAGATCGCCGTTGCCTCTGGTCAAGATAACGGGCGAAAGTCGGCGGGGGGGAGGCCGT  1378
         |  | | ||      | |   || ||  | || || |             |     |       |    ||| ||||    |  |
Def-B   ACACCGTGATGTGACAGGACCCCCCCTTAATCAACCTCAGTGGGATCACAACTTGGCTGAAGGTAAGGAACGTCGCCTAGTCT--ACCA-------------------------------  1637

KoRV-B  CCGCTCCTTCCCGGCTCCCCATCTACCCGGAGACGGACAGCCTGTTCCTCCTCTCAGAACCCCCGCCCTATCCAACATCCCCTCCACCTCCCCCAGCCCCTCACGCGGCCAGACCAGCGC  1498

Def-B   ------------------------------------------------------------------------------------------------------------------------  1637

KoRV-B  CAGGCCTAATGGCTGAGGGACTCGGCTCTGAGGGACCGGCCGCTGGAACTAGGAGTCGCCGTCCCCGCAGTCCTACGGGTGACACTGGTCCTGACTCCACTGTGGCCCTGCCCCTCCGGG  1618

Def-B   ------------------------------------------------------------------------------------------------------------------------  1637

KoRV-B  CCGTGGGACCCCCGGCCGAACCAAACGGCTTAGTCCCCTTGCAATATTGGCCTTTCTCCTCAGCAGATCTCTATAATTGGAAATCTAATCATCCTTCTTTTTCCGAGAATCCTACAGGAC  1738

Def-B   ------------------------------------------------------------------------------------------------------------------------  1637

KoRV-B  TCACGGGACTCCTTGAGTCCCTCATGTTTTCCCACCAGCCTACTTGGGACGATTGCCAGCAGCTCCTGCAAGTCCTCTTTACCACCGAGGAAAGAGAAAGGATTCTTTTGGAGGCTCGCA  1858

Def-B   ------------------------------------------------------------------------------------------------------------------------  1637
```

```
KoRV-B   AGAACGTCCTTGGAGTTAACGGGGCCCCTACACAACTCGAGAACCTCATTAATGAGGCCTTCCCCCTTAATCGACCTCAATGGGATCACAACACGGCTGAAGGTAGGGAGCGTCTCCTGG  1978

Def-B    ------------------------------------------------------------------------------------------------------------------------  1637

KoRV-B   TCTACCGCCGGACTCTAGTGGCGGGTCTCAAAGGGGCAGCAAGGCGCCCCACCAATTTGGCCAAGGTAAGAGAAGTCTTGCAGGGACCAACAGAACCCCCGTCCGTTTTTCTAGAACGTC  2098
                |||||||||||||||||||||||||||||||  |||||||||||||||||||||||||||| ||||  ||||| || ||||||| | | |||||||||||||||| |||||||
Def-B    -------CCGGACTCTAGTGGCGGGTCTCAAAGGGGCAAAAAGGCGCCCCACCAATTTGGCCAAGGTAGGAGAGATCTTGTAGAGACCAACGGGATCCCCGTCCGTTTTTCTTGAACGTC  1750

KoRV-B   TAATGGAGGCTTATAGGAGATATACCCCATTTGATCCCTCTTCTGAGGGACAAAAGGCGGCGGTGGCCATGTCCTTTATCGGACAGTCTGCCCCGGATATTAAAAAGAAATTACAAAGAC  2218
         ||| ||        ||||||||||||||||             |       ||| ||||  ||||||| |||||||||||||||||||
Def-B    TAACGG--------AGGAGATATACCCCAT------------AT-GATACAAAATGGCGCTGGTGGCCTTGTCCTTTATCGGACAGTC--------------------------------  1817

KoRV-B   TGGAAGGACTCCAGGACCATTCCTTGCAGGATCTCATAAAGGAGGCAGAGAAGGTATATCATAAGAGAGAGACAGAGGAAGAAAAGCAAGAAAGAGAGAAAAAGGAGACAGAGGAAAGAG  2338

Def-B    ------------------------------------------------------------------------------------------------------------------------  1817

KoRV-B   AGAGACGGCGTGACAGGCGCCAGGAGAAAAACTTGACCAAAAT--CTTGGCCGCGGTAGTAAGTGAGAAAGGGTCCAGAGGGAGACAGGCAGGGAATCTGAGCAACAGGGCAATGAGGGC  2456
                                                      | ||       |    |      |  |     | ||    ||||    |      ||  |  |  |   |
Def-B    -----------------------------------------CGCCCCTGATATTACT-AAGAAATCACAGAGACTGGGGGGACTCCAGGACCAGGGAGGCAGGTAGGGACCTGAGCAACA  1895

KoRV-B   ACCTAGGGAAGGAAGACCGCCTCTGGACAAAGATCAGTGCGCATACTGTAAAGAAAGGGGCCATTGGGCAAGGGAGTGCCCCCGGAAGAAGAACGCCAGGGAAACCAATGTCCTGACCCT  2576
         |   |  ||      |  |      |                   | ||    | | ||     || ||||| ||||| ||| |||||||| ||| |||| || |||  ||||||||| |
Def-B    AGGCAATGAGCACCTAAGGAAGAAAGGTCGCCTCTGCACGAAGACCAGTGTGCATATGGAAAGCTGAGCAAGAGAGTGTCCCTGGAAGAAGTACGGCAGGAAAGCCAGAGTCCTGACCTT  2015
                     Primer P2
               gag]
KoRV-B   GGGTGACTAGGGGAGTCGGGGTTCGGACCCCCTCCCCGAGCCCAGGGTAACGTTAACTGTGGAGGGGATCCCCACTGAGTTTTTGGTTGATACCGGGGCCGAACATTCAGTATTGACCAA  2696
         ||| |||||]|| |    |  |    |||    | | ||        |   |       ||||    | ||      | |          |  | |     || |    || |     ||
Def-B    GGGCGACTAAGGAAAGTTGCCTAGTAACCAGGACACTGACGGGCATATTTTGGGTGAATTGAAGTTTACCCTACAGAAATAAAGACAGCCTGACCGTCAGCAAGAAGATGTTTGAGGGAA  2135

             [Pro-
KoRV-B   GCCTATGGGAAAGATGGGACCCAAACGGACAGTCGTGGCTGGGGCGACGGGCAGCAAAGTTTACCCTTGGACCACCAAGAGACTTTTGGAAATTGGACAAAAGCAAGTGACCCACTCATT  2816
          | |[
Def-B    TCTTACCTCGCTTT----------------------------------------------------------------------------------------------------------  2149

KoRV-B   CTTGGTCATACCCGAATGCCCTGCTCCCCTGTTAGGCAGGGACCTCCTCACCAAGCTAAAAGCTCAAATCCAGTTTTCCACAGAGGGCCCACGGGTAACATGGGAAGACCGCCCCGCCAT  2936

Def-B    ------------------------------------------------------------------------------------------------------------------------  2149

KoRV-B   GTGTTTAGTCCTGAACTTGGAGGAGGAATACCGATTACACGAAAAGCCGgTCCCTCCTTCTATCGACCCGTCATGGCTCCAACTCTTTCCCATGGTTTGGGCCGAGAAGGCAGGTATGGG  3056

Def-B    ------------------------------------------------------------------------------------------------------------------------  2149
```

```
KoRV-B  ACTGGCCAATCAAGTCCCACCAGTGGTAGTGGAACTGAAGTCAGATGCCTCACCAGTGGCTGTTCGACAGTACCCAATGAGCAAGGAAGCCCGGGAGGGTATCAGGCCCCACATCCAAAG  3176

Def-B   ------------------------------------------------------------------------------------------------------------------------  2149

KoRV-B  ATTCTTGGATTTGGGGATTCTGGTACCTTGCCAGTCGCCCTGGAACACCCCTTTGTTACCTGTAAAAAAGCCTGGAACCAATGACTATCGGCCAGTCCAAGATTTGAGGGAGGTCAACAA  3296

Def-B   ------------------------------------------------------------------------------------------------------------------------  2149

KoRV-B  AAGAGTACAGGACATTCATCCCACAGTCCCGAACCCCTATAACCTACTCAGTTCCCTCCCGCCCAGCCACACCTGGTATTCAGTTTTAGACCTCAAAGATGCTTTCTTTTGCCTCAAGCC  3416

Def-B   ------------------------------------------------------------------------------------------------------------------------  2149

KoRV-B  ACACCCCAACAGCCAGCCATTGTTCGCGTTTGAATGGAGAGATCCAGAAAAAGGCAATACTGGCCAGCTAACCTGGACTCGGCTACCGCAAGGATTCAAGAACTCTCCCACCCTCTTCGA  3536

Def-B   ------------------------------------------------------------------------------------------------------------------------  2149

KoRV-B  TGAGGCCCTCCACCGGGATCTGGCATCCTTCAGGGCTCTCAACCCCCAGGTAGTGATGCTCCAATATGTTGATGACCTCCTGGTAGCGGCCCCAACGTACCGAGACTGCAAAGAAGGGAC  3656

Def-B   ------------------------------------------------------------------------------------------------------------------------  2149

KoRV-B  ACGAAGGCTCTTACAGGAACTAAGTAAGTTGGGATACCGAGTGTCGGCCAAAAAGGCCCAGCTTTGCCGGGAGGAAGTTACGTATCTCGGGTATTTGCTAAAAGGGGGGAAGAGATGGCT  3776

Def-B   ------------------------------------------------------------------------------------------------------------------------  2149

KoRV-B  GACCCCAGCCCGGAAGGCCACTGTAATGAAGATCCCCACCCCTACAACCCCCAGACAAGTCCGTGAGTTTTTGGGCACTGCTGGATTCTGTAGGCTCTGGATCCCTGGTTTCGCTTCCCT  3896

Def-B   ------------------------------------------------------------------------------------------------------------------------  2149

KoRV-B  GGCTGCACCCCTGTACCCCCTGACGAGAGAAAAGGTTCCCTTTACCTGGACTGAGGCCCACCAGGAAGCCTTTGGCCGTATAAAAGAGGCCCTGCTATCGGCCCCCGCCCTGGCCCTTCC  4016

Def-B   ------------------------------------------------------------------------------------------------------------------------  2149

KoRV-B  GGACCTCACCAAGCCATTTGCTCTGTACGTGGATGAAAAAGAGGGTGTTGCCCGGGGGGTGCTTACTCAAACCCTAGGACCATGGCGACGCCCGGTAGCTTACCTGTCAAAAAAATTAGA  4136

Def-B   ------------------------------------------------------------------------------------------------------------------------  2149

KoRV-B  TCCGGTGGCCAGTGGGTGGCCAACTTGCCTAAAAGCAATTGCGGCCGTAGCACTCCTTCTCAAGGACGCTGATAAGTTAACCCTAGGACAGAACGTGTTGGTGATTGCACCTCACAACCT  4256

Def-B   ------------------------------------------------------------------------------------------------------------------------  2149
```

```
KoRV-B  GGAAAGTATTGTGAGACAGCCCCCCGACCGATGGATGACAAATGCCAGGATGACCCATTACCAAAGTCTGCTGCTGAATGAGAGGGTGTCTTTTGCGCCCCCGGCCATTCTGAATCCTGC  4376

Def-B   ------------------------------------------------------------------------------------------------------------------------  2149

KoRV-B  CACCCTGCTCCCCGTAGAGTCAGACGACACCCCAATACACATATGCTCAGAAATCCTTGCCGAGGAGACAGGAACCCGACCTGATCTGAGAGATCAGCCGTTGCCCGGGGTACCCGCTTG  4496

Def-B   ------------------------------------------------------------------------------------------------------------------------  2149

KoRV-B  GTATACGGATGGCAGCAGTTTCATCATGGATGGCAGGCGACAAGCAGGTGCTGCCATTGTGGACAACACGCGGACGGTGCGGGCGAGCAACCTGCCAGAGGGAACGTCAGCTCAGAAGGC  4616

Def-B   ------------------------------------------------------------------------------------------------------------------------  2149

KoRV-B  CGAACTAATCGCCTTGACACAAGCATTACGCTTGGCCGAAGGAAAAAGCATCAACATCTACACGGACAGCAGGTACGCTTTTGCCACCGCTCATGTTCACGGGGCTATATACAAACAAAG  4736

Def-B   ------------------------------------------------------------------------------------------------------------------------  2149

KoRV-B  AGGACTGCTCACTTCCGCTGGAAAAGACATCAAGAACAAAGAGGAGATTTTGGCCCTGCTAGAGGCCATCCATCTCCCTAAGCGAGTCGCTATCATCCATTGCCCCGGCCACCAAAGAGG  4856

Def-B   ------------------------------------------------------------------------------------------------------------------------  2149

KoRV-B  GACTGACCCTGTGGCTACCGGAAACCGAAAGGCCGATGAGGCCGCGAAACAAGCTGCCCAGTCAACCAGAATACTGACGGAGACCACGAAAAATCAAGAACACTTCGAGCCTACACGGGG  4976

Def-B   ------------------------------------------------------------------------------------------------------------------------  2149

KoRV-B  GACGATCAAGCCGAAAGAGCTCACCCCTGACCAGGGAAGGGAATTCATTCAACGGCTGCATCAGCTCACGCACTTAGGGCCAGACAAGCTTCTCCAGCTTGTAGGTCGCACCAGTCTTCA  5096

Def-B   ------------------------------------------------------------------------------------------------------------------------  2149

KoRV-B  CATCCCGAATCTCCAGTCTGTAGTGCGTGAAATCACCAGCAAGTGTCAGGTTTGTGCTGTAACTAACGCAGTCACCACCTACCGAGAGTCTGGAAGGAGGCAAAGGGGAGATCGACCCGG  5216

Def-B   ------------------------------------------------------------------------------------------------------------------------  2149

KoRV-B  CGTGTATTGGGAAGTAGACTTCACAGAGGTTAAGCCTGGCCGGTATGGTAATAGATATTTGCTAGTGTTTATCGATACATTTTCTGGGTGGGTAGAAGCTTTCCCTACAAAAACAGAGAC  5336

Def-B   ------------------------------------------------------------------------------------------------------------------------  2149

KoRV-B  AACCCTGACCGTCTGCAAGAAGATACTTGAGGAAATCCTACCTCGTTTCGGGATCCCTAAGGTACTCGGGTCCGACAATGGCCCTGCCTTCGTTGCTCAGGTAAGCCAGGGGCTGGCCAC  5456
                                                         ||||||||||||||||||||||||||| ||| |||| |||||||||||||||||||||||||| |||||||
Def-B   -------------------------------------------------GGGATCCCTAAGGTACTCGGGTCCGACCATGACCCTACCTTCGTTGCTCAGGTAAGCCAGGGGTTGGCCAC  2220
```

```
KoRV-B   TCAACTGGGGATTAATTGGAAATTACATTGTGCATATAGACCCCAGAGCTCAGGTCAGGTAGAAAGGATGAATAGGACAACTAAAGAGACCTTGACAAAATTGGCCTTAGAGACCGGTGG  5576
Def-B    TCAACTGGGGATTGATTGGAAATTACATTGTGCATATAGACCCCAGAGCTCAGGTCAGGTAGAAAGGAAGAATAGGACAATTAAAGAGGCCCTGACAAAATTGGCCTTAGAGACAGGCGG  2340

KoRV-B   AAAAGACTGGGTGACCCTCCTCCCCTTGGCGCTACTTAGGGCCCGGAGTACCCCTGGCCAGTTTGGCCTGACTCCTTATGAGATTCTTCATGGGGGGCCGCCCCCTGTACTTGCGTCTGG  5696
Def-B    AAAAGACTGGGTGACCCTCCTCCC-TTT-GGCG-CTGCTTAGGGCCTGGAATACCCTGACTCTTCATGAGATTCTTCAAGAAGG-------GGGGGGCCGCCCCCTGTACTTGTGTCTGG  2450

KoRV-B   AGAAGTCGTGGGTTCTAATGGTGATTTTTTTCCCGTTCTATTTACCCACTTGAAGGCTTTAGAGGTTGTGAGGACTCAGATCTGGGACCAGATAAAAGAAGCCTACAGGCCTGGTACTGT  5816
Def-B    GGAAGTCGTGGGTTCTAATGATGATTTTTTTTCCGTTCTATTCACCCACATGAAAGCTTTAGAGGTTGTGAGGACTC--------GACCAGATAAAAGAAGCCTCCAGGCTTGGTACTGT  2562

KoRV-B   GGCCATTCCCCACCCGTTCCAGGTCGGTGACCGAGTGCTGGTTAGACGTCATCGGTCCGGCAGCCTTGAGCCTCGGTGGAAAGGCCCATACCTGGTGCTACTGACCACCCCGACCGCGGT  5936
Def-B    GACCATTCCCCACCCGTTCCAGGTCGGTGACCAAGTGCTGGTTAGACGCCATCGGTCCGGCAGCCTTGAGCCTCGGTGGAAAGGCTCATACCTGGTGCAA--------------------  2663

                                       env
KoRV-B   GAAGGTTGACGGTATTGCTGCCTGGGTCCATGCTTCTCATCTCAAACCCGCGCCACCTGGGGC-ACCCGATGAGTCCTGGGAACTGGAAAAGACTGATCATCCTCTTAAGTTGCGTGTTCG  6056
Def-B    ----------------------------C-TGCTTCTCATCTCAAACCCGCGCCACCTGGGGTACCCGATGAGTCCTGGGAACTGGAAAGGACTGATCATCCTCTTAAGTTGTGTGTTCG  2753

                                    Pro-pol
KoRV-B   GCGGCGCCGAAATGAATCAACAGCATAACAACCCTCACCAACCCATGACTCTCACCTGGCAGGTACTGTCCCAGACGGGAAGTGTCGTTTGGGAAAAGAAAGCAGTCGAGCCACCCTGGA  6176
Def-B    GCGGCGCCGAAATGTATCAACAGCAT---AACCCTCACCAACCCATGACTCTCACCTGGCAGGTACTGTCCCAGACGGGAAGCGTCGTTTGGGAAAAGAAAGCAGTCGAGCCACCCTGGA  2870

KoRV-B   CGTGGTGGCCCTCTCTTGAGCCTGATGTGTGTGCGCTGGCGGCGGGTCTTGAGACCTGGGGCATCCCGCACCTCACCGCGCCAGAATCTCAACAGTCTGCGCCCCCCGATGGCTGTGGGG  6296
Def-B    CGTGGTGGCCCTCACTTGAGCCTGATGTGTGTGCGCTGGCGGCGGGTCTTGAGACCTGGGGCATCCCGCACCTCACCGCGCCAGAATCTCAACAGTCTGCGCCCCCCGATGGCTGTGGGG  2990

KoRV-B   TGCTTTATAGTCAGGTCGGCAGAGGGTTGGTCAGGCAATCGAGCTATGGAACCTTGGGGTGTCGCTGTCCCCGAGACCGGAACAGACTGGCTCAATCTCAGTTCTATGTGTGTCCCCGAG  6416
Def-B    TGCTTTATAGTCAGGTCGGCAGGGGGTTGGTCAGGCAATCGAGCTATGGAACCTTGGGGTGTCGCTGTCCCCGAGACCGGAACAGACTGGCTCAATCTCAGTTCTATGTGTGTCCCCGGG  3110
                                                                                  Primer P3 complementary

KoRV-B   ATGGCCGTTCCCTGTCCGAGGCCTGGAGGTGCGGAGGGTTTGAGTCTTTGTATTGTAAAGAGTGGGGTTGTGAGACCACGGGAACCGCCTACTGGCAACCCCGGTCCTCGTGGGACCTTA  6536
Def-B    ATGGCCGTTCCCTGTCCGAGGCCTGGAGGTGCGGAGGGTTTGAGTCTTTGTACTGTAAAGAGTGGGGTTGTGAGACCACGGGAACCGCCTACTGGCAACCCCGGTCCTCGTGGGACCTTA  3230

KoRV-B   TAACTGTAGGCCTGGGCCACCCTACGGGGACATGCGAACACACCGGTTGGTGCAACCCACTCAAGATAGAGTTCACCGAGCCTGGCAAACGGTTCCGAAACTGGCTACAGGGGCGAACCT  6656
Def-B    TAACTGTAGGCCGGGGCCACCCTACGGGGACATGCGAACACACCGGTTGGTGCAACCCACTCAAGATAGAGTTCACCGAGCCTGGCAAACGGTTCCGAAACTGGCTACAGGGGCGAACCT  3350
```

```
KoRV-B  GGGGTCTAAGGTTCTATGTGACTGGACACCCAGGCGTGCAGTTGACCATACGACTAGTGATCACAAGTCCCCCACCGGTAGTAGTGGGCCCCGATCCTGTCCTCGCAGAACAAGGACCTC  6776
        |||||||||||||||||||||||||||||||||||||||||||||||||||||||||||||||||||||||||||||||||||||||||||||||||||||||||||| |||||||||||
Def-B   GGGGTCTAAGGTTCTATGTGACTGGACACCCAGGCGTGCAGTTGACCATACGACTAGTGATCACAAGTCCCCCACCGGTAGTAGTGGGCCCCGATCCTGTCCTCGCAGGACAAGGACCTC  3470

KoRV-B  CCAGAAAAATTCCCTTCCTCCCCAGAGTGCCGGTTCCTACCCTATCACCCCCAGCGTCCCCTATCCCCACGGTACAGGCTAGCCCCCCGGCCCCTAGTACTCCATCTCCCACCACGGGCG  6896
        ||||||||||||||||||||||||||||||||||||||||||||||||||||||||||||||||||||||||||||||||||||||||||||||||||||||||||||||||||||||||
Def-B   CCAGAAAAATTCCCTTCCTCCCCAGAGTGCCGGTTCCTACCCTATCACCCCCAGCGTCCCCTATCCCCACGGTACAGGCTAGCCCCCCGGCCCCTAGTACTCCATCTCCCACCACGGGCG  3590

KoRV-B  ACCGGCTCTTTGGCCTCGTACAAGGGGCCTTTCTGGCCTTGAATGCCACCAACCCGGAGGCCACGGAGTCTTGCTGGCTCTGTCTAGCCCTCGGCCCTCCTTATTACGAAGGAATCGCAA  7016
        ||||||||||||||||||||||||||||||||||||||||||||||||||||||||||||||||||||||||||||||||||||||||||||||||||||||||||||||||||||||||
Def-B   ACCGGCTCTTTGGCCTCGTACAAGGGGCCTTTCTGGCCTTGAATGCCACCAACCCGGAGGCCACGGAGTCTTGCTGGCTCTGTCTAGCCCTCGGCCCTCCTTATTACGAAGGAATCGCAA  3710

KoRV-B  CTCCAGGGCAAGTCACTTATGCCTCCACAGATTCCCAATGTCGCTGGGGAGGAAAAGGGAAGCTCACCCTCACTGAAGTCTCTGGACTCGGGTTATGTATAGGGAAAGTACCTCCTACTC  7136
        ||||||||||||||||||||||||||||||||||||||||||||||||||||||||||||||||||||||||||||||||||||||||||||||||||||||||||||||||||||||||
Def-B   CTCCAGGGCAAGTCACTTATGCCTCCACAGATTCCCAATGTCGCTGGGGAGGAAAAGGGAAGCTCACCCTCACTGAAGTCTCTGGACTCGGGTTATGTATAGGGAAAGTACCTCCTACTC  3830

KoRV-B  ACCAACATCTCTGCAACCTGACTATACCCTTAAACGCCTCACATACTCACAAGTACCTCCTCCCCTCCAACCACAGCTGGTGGGCTTGTAATTCTGGCCTCACCCCCTGCCTCTCTACAT  7256
        ||||||||||||||||||||||||||||||||||||||||||||||||||||||||||||||||||||||||||||||||||||||||||| |||||||||||||| |||||||||||||
Def-B   ACCAACATCTCTGCAACCTGACTATACCCTTAAACGCCTCACATACTCACAAGTACCTCCTCCCCTCCAACCACAGCTGGTGGGCTTGTAATACTGGCCTCACCCCATGCCTCTCTACAT  3950

KoRV-B  CAGTCTTCAACCAGTCTAACGATTTCTGTATCCAGATCCAGCTTGTCCCTCGCATCTACTATCACCCAGACGGTACCTTGCTACAGGCCTATGAGTCCCCCCACCCTAGAAACAAGAGAG  7376
        ||||||||||||||||||||||||||||||||||||||||||||||||||||||||||||||||||||||||||||||||||||||||||||||||||||||||||||||||||||||||
Def-B   CAGTCTTCAACCAGTCTAACGATTTCTGTATCCAGATCCAGCTTGTCCCTCGCATCTACTATCACCCAGACGGTACCTTGCTACAGGCCTATGAGTCCCCCCACCCTAGAAACAAGAGAG  4070

KoRV-B  AGCCTGTCTCACTCACCCTGGCTGTCCTTCTCGGATTAGGGGTCGCAGCAGGTATAGGTACCGGCTCGACCGCCCTAATAAAAGGGCCCATAGACCTCCAACAAGGTTTGACTAGCCTCC  7496
        |||||||||||||||| |||||||||||||||||||||||||||||||||||||||||||||||||||||||||| ||||| ||||||||||||||||||||||
Def-B   AGCCTGTCTCACTCACTCTGGCTGTCCTTCTCGGATTAGGGGTCGCAGCAGGTATAGGTACCGGCTCGACCGCCCTAATAAAAGGGCCCATGGACCTACAACAAGGTTTGACTAGCCTCC  4190

KoRV-B  AAATTGCCATGGATACAGACCTTAGAGCCCTTCAAGACTCCGTAAGTAAACTAGAGAATTCCTTAACCTCCCTTTCTGAAGTAGTGCTCCAGAATAGGAGAGGCCTTGATCTGCTATTTT  7616
        ||||||||||||||||||| ||||||||||||||||||||||||||||||||||||||||||||||||||||||||||||||||||||||||||||||||||||||||||||||||||||
Def-B   AAATTGCCATGGATACAGATCTTAGAGCCCTTCAAGACTCCGTAAGTAAACTAGAGAATTCCTTAACCTCCCTTTCTGAAGTAGTGCTCCAGAATAGGAGAGGCCTTGATCTGCTATTTT  4310

KoRV-B  TGAAGGAAGGGGGCCTTTGTGCAGCCCTAAAAGAGGAGTGCTGTTTCTATGTTGACCACTCAGGCGCGGTGCGAGACTCCATGAGGAGACTCAAGGAAAGGTTAGATAAGAGGCAGTTAG  7736
        ||||||||||||||||||||||||||||||| ||||||||||||||||||||||||||||||||| ||||||||||||||||||||| ||||||||||||||||||||||||||||||||
Def-B   TGAAGGAAGGGGGCCTTTGTGCAGCCCTAAAGGAGGAGTGCTGTTTCTATGTTGACCACTCAGGCCCGGTGCGAGACTCCATGAGGAAACTCAAGGAAAGGTTAGATAAGAGGCAGTTAG  4430

KoRV-B  AGCACCAAAAGAATTTAAGTTGGTACGAGGGATGGTTCAACCGTTCCCCCTGGCTTACTACTTTACTGTCTGCCCTTGCTGGTCCCCTGCTACTCCTCCTTCTG-TTACTCACCCTCGGG  7855
        |||||||||||||||||||||||||||||||||||||||||||||||||||||||||||||||||||||||||||||||||||||||||||||||||||||||| || ||||||||||||
Def-B   AGCACCAAAAGAATTTAAGTTGGTACGAGGGATGGTTCAACCGTTCCCCCTGGCTTACTACTTTACTGTCTGCCCTTGCTGGTCCCCTGCTACTCCTCCTTCTGATT-CTCACCCTCGGG  4549
```

```
                                                                                                                                                          env]
KoRV-B   CCTTGTGTCATCAATAAGTTAGTGCAATTCATCAATGATAGGGTTAGTGCAGTAAGGATTCTGGTTCTCAGGCACAAGTACCAGACCCTAGACAACGAGGATAACCTTTAATTCTGCTCT   7975
         |||||||||||||||||||||||||||||| ||||||||||||||||||||||||| |||||| | ||||| ||||||||||||||||||||||||||||||||||||||||||||||||
Def-B    CCTTGTGTCATCAATAAGTTAGTGCAATTCGTCAATGATAGGGTTAGTGCAGTAAGAATCCTGTTCCTCAGACACAAGTACCAGACCCTAGACAACGAGGATAACCTTTAATTCTGCTCT   4669

KoRV-B   ATGATTAGAGCTACCCTCAAAGAAAATGGGGGAATGAAGGAGGCAGAAATCATGAGGCAGAAATCATTCCGTGAAGTATGGAAACTACCCGGAGGGCCCCAGGGTTAGGGACAAGCGCAG   8095
         ||||||||||||||||||||||||||||||||||||||||||||||||||||||||||||||||||||||||||||||||||||||||||||||||||||||||||||||||||||||||
Def-B    ATGATTAGAGCTACCCTCAAAGAAAATGGGGGAATGAAGGAGGCAGAAATCATGAGGCAGAAATCATTCCGTGAAGTATGGAAACTACCCGGAGGGCCCCAGGGTTAGGGACAAGCGCAG   4789

KoRV-B   CCAGGCACAGTAAAAGGTCAGAGCAAGAAAAACAAGGAAGGTTTGGAGTGCCAAACGGAATATCTGTGGTCAACGGAATATCTGTGG… 8215
         |||||||||||||||||||||||||||||||||||||||||||||||||||||||||||||||||||||||||||||||||||||||
Def-B    CCAGGCACAGTAAAAGGTCAGAGCAAGAAAAACAAGGAAGGTTTGGAGTGCCAAACGGAATATCTGTGGTCAACGGAATATCTGTGG  4876
                                                                        Primer P7 complementary
```

Supplemental table 1. Host range of KoRV variants.

	KoRV-A	KoRV-B	KoRV-C	KoRV-D
MDTF-THTR1	-*	+**	-	-
MDTF-PiT1	+	-	-	-
XC	+	-	-	-
BAT	+	+	+	-
HOS	+	+	+	-
HT1080	+	+	+	-
HT1080-KAZAP	-	+	+	-
HT1080-KBZAP	+	-	+	-

- Denotes a normalized value of < 0.01
- \+ denotes a normalized value of 1
